# Isolation, Characterization, and Tea Growth-Promoting Analysis of JW-CZ2, a Bacterium With 1-Aminocyclopropane-1-Carboxylic Acid Deaminase Activity Isolated From the Rhizosphere Soils of Tea Plants

**DOI:** 10.3389/fmicb.2022.792876

**Published:** 2022-02-28

**Authors:** Hui Liu, Guang-Hui Chen, Jing-Jing Sun, Shu Chen, Yong Fang, Jia-Hong Ren

**Affiliations:** ^1^School of Ecology and Environment, Anhui Normal University, Wuhu, China; ^2^Anhui Provincial Engineering Laboratory of Water and Soil Pollution Control and Remediation, Wuhu, China; ^3^College of Biosystems Engineering and Food Science, Zhejiang University, Hangzhou, China; ^4^Department of Life Sciences, Changzhi University, Changzhi, China

**Keywords:** plant growth promoting rhizobacteria, tea plant, ACC deaminase, genome sequencing, *Serratia marcescens*

## Abstract

One of the major mechanisms underlying plant growth-promoting rhizobacteria (PGPR) is the lowering of ethylene level in plants by deamination of 1-aminocyclopropane-1-carboxylic acid (ACC) in the environment. In the present study, using ACC as the sole nitrogen source, we screened seven ACC deaminase-producing bacterial strains from rhizosphere soils of tea plants. The strain with the highest ACC deaminase activity was identified as *Serratia marcescens* strain JW-CZ2. Inoculation of this strain significantly increased shoot height and stem diameter of tea seedlings, displaying significant promotive effects. Besides, *S. marcescens* strain JW-CZ2 displayed high ACC deaminase activities in wide ranges of ACC concentration, pH, and temperature, suggesting the applicable potential of JW-CZ2 as a biofertilizer. Genome sequencing indicated that clusters of orthologous groups of proteins (COG) annotation and Kyoto Encyclopedia of Genes and Genomes (KEGG) pathways of JW-CZ2 mainly included amino acid transport and metabolism, transcription, carbohydrate transport and metabolism, inorganic ion transport and metabolism, and membrane transport. Moreover, genes in relation to phosphate solubilization, indole acetic acid (IAA) production, and siderophore were observed in the genome of JW-CZ2, and further experimental evidence demonstrated JW-CZ2 could promote solubilization of inorganic phosphate, inhibit growth of pathogenic fungi, and produce IAA and siderophore. These aspects might be major reasons underlying the plant growth-promoting function of JW-CZ2. Overall, this study provides a new *S. marcescens* strain, which has applicable potential as a promising biofertilizer.

## Introduction

The tea plant (*Camellia sinensis* L. O. Kuntze) is an evergreen perennial shrub and is broadly cultivated in the tropical and subtropical regions across the world (Wang et al., 2016). The young leaves of the tea plant are processed to prepare tea for drinking. China is the source of indigenous tea plants and the birthplace of cultivated tea plantation (Liu et al., 2019). However, the productivity of tea is decreasing in recent years. The underlying reasons include nutrient deficiency and abiotic stresses. The annual plucking of new shoots easily induces nutrient deficiency of soils (Upadhyaya and Panda, 2013). To supplement this, more chemical fertilizers are applied, which causes nutrient imbalance and soil pollution and finally negatively affects tea plantations. Thus, alternative environmentally friendly fertilizer or technology is required.

In the rhizosphere of plants, a broad spectrum of bacteria reveal promotive effects on plant growth, yield, and resistance to environmental stresses, which are named plant growth-promoting rhizobacteria (PGPR) (Numan et al., 2018). As reported, PGPR could promote plant growth and increase the tolerance to stresses in plants. In addition, the application of PGPR generally does not cause environmental pollution, contributing significantly to sustainable agriculture (Figueiredo et al., 2016; Ramakrishna et al., 2019). Thus, PGPR are more environmental friendly and an ideal alternative to replace chemical fertilizers.

Various mechanisms underlying the promotion of PGPR for plant growth have been reported, depending on the species of the plant and the bacterium, including enhancement of nitrogen fixation (James and Baldani, 2012), phosphate solubilization (Calvo et al., 2017), induction of siderophore (Ramakrishna et al., 2019), production of indole acetic acid (IAA) (Pérez-Flores et al., 2017), competitive exclusion of pathogens (Ahemad and Kibret, 2014), stimulation of mycorrhizae development, and removal of phytotoxic substances (Bashan and de-Bashan, 2010). More specifically, ethylene is a ubiquitous plant hormone and plays multiple roles in the regulation of plant growth and development. Under adverse environment, the production of ethylene was stimulated to increase plant resistance but restrain the growth and the final biomass of plants (Tiwari et al., 2018). Some PGPR revealed a high activity of 1-aminocyclopropane-1-carboxylic acid (ACC) deaminase, which can hydrolyze ACC to α-ketobutyrate (α-KB) and ammonia and then further utilize them as a carbon and nitrogen source (Penrose and Glick, 2003; Tiwari et al., 2018; Gupta and Pandey, 2019; Nascimento et al., 2019). This kind of PGPR is called ACC deaminase-producing bacterium (ADB), which has been reported in *Agrobacterium*, *Arthrobacter*, *Azotobacter*, *Azospirillum*, *Bacillus*, *Burkholderia*, *Caulobacter*, *Chromobacterium*, *Erwinia*, *Flavobacterium*, *Micrococcous*, *Pseudomonas*, and *Serratia* (Bhattacharyya and Jha, 2012). Since ACC is an immediate precursor of ethylene, reduction of ACC could inhibit the production of ethylene and finally alleviate the inhibitory effects of ethylene on plant growth (Li et al., 2000; Glick, 2014; Gupta and Pandey, 2019). Moreover, utilization of ADB revealed enhancive effects on the growth of many crops and reduced susceptibility to diseases caused by plant pathogens under either stressful or normal conditions, displaying applicable and commercial values of PGPR as biofertilizer on crop plantation. For example, field application of *Bacillus* PSB12, PSB5, and *Enterobacter* 77-NS5 significantly increased dry weight and length of root and shoot, as well as seed weight in wheat (Mukhtar et al., 2017). Inoculation of *Sinoorhizobium meliloti*, *Bacillus flexus* and *Bacillus megaterium* separately or together in P-deficient soils improved plant height, shoot and root dry weight, as well as P nutrition in maize (Ibarra-Galeana et al., 2017). Fertilization of *Bacillus subtilis* increased growth and salt tolerance in *Arabidopsis* (Cabrefiga et al., 2007; Han et al., 2014), *Bacillus amyloliquefaciens* could effectively prevent bottom rot in lettuce (Chowdhury et al., 2013), and *Pseudomonas fluorescens* A506 could control fire blight in pome fruits by competing with the pathogen *Erwinia amylovora* (Cabrefiga et al., 2007).

Generally, the promotive effects of PGPR on plant growth are affected by plant species, cultivar, genotype, and soil type (Lucy et al., 2004). However, some reports also indicated that PGPR strains isolated from one plant species could excellently promote growth of another plant species (Aggangan and Anarna, 2019; Mayer et al., 2019; Gou et al., 2020). Theoretically, native PGPR strains may be more adaptative to the local environment than non-native strains, and thus may show better promotive functions on local target plants (Fischer et al., 2007). In the rhizosphere soil of tea plants, abundant microbial resources have been identified (Chahboune et al., 2011; Zhao et al., 2012; Wang and Han, 2019). However, up to date, investigations of ADB from rhizosphere of tea plants are still limited. In the present study, we collected rhizosphere soils from seven tea plantation gardens in the Anhui province, China. From these samples, ADB were screened and their ACC deaminase activities were compared. The strain with the highest activity was then applied on tea plants to evaluate their effects on tea growth. Afterward, the effects of culture conditions on ACC deaminase activity were assayed to evaluate its applicability on fields. To further explore the mechanisms underlying its plant growth-promoting functions, the whole genome of this strain was sequenced, and the predicted potential mechanisms were further validated by experimental evidence. Overall, these results may contribute a novel plant growth-promoting bacterial strain to tea plantation and enrich our understandings in the mechanisms underlying the promotion of PGPR on plant growth.

## Materials and Methods

### Isolation of 1-Aminocyclopropane-1-Carboxylic Acid Deaminase-Producing Bacteria From Rhizosphere Soil of Tea Plant

The rhizosphere soil samples of tea plants were collected from seven different tea gardens in Anhui Province, the south of Yangtze river region, China (Supplementary Table 1). ADB were enriched and then isolated as described in Penrose and Glick (2003) with modifications. Briefly, 1 g of rhizosphere soil was suspended in 50 ml of sterile Dworkin and Foster (DF) salts minimal medium (Dworkin and Foster, 1958) and then cultured in a shaker at 200 rpm and 28°C. After 24 h, 1 ml of the liquid was transferred to a 250-ml flask containing 50 ml of sterile DF salts minimal medium, in which 3.0 mM ACC was supplied instead of (NH_4_)_2_SO_4_ as the sole nitrogen source. After being further cultured for 24 h, the medium was diluted, spread on DF salts minimal medium agar plates, and cultured for 48 h at 28°C. The bacterial colonies were picked and stored in 20% glycerol (v/v) solution at –80°C for further experiments.

### Analysis of 1-Aminocyclopropane-1-Carboxylic Acid Deaminase Activity

Selected bacterial strains were cultured in tryptic soybean broth (TSB) medium at 28°C and 200 rpm for 12 h (approximately 3 × 10^8^–5 × 10^8^ CFU/ml) and washed twice with sterile DF salts minimal medium. The harvested cells were transferred to the DF salt minimal medium containing 3 mM ACC [instead of (NH_4_)_2_SO_4_] and cultured at 28°C and 200 rpm for 24 h to induce the production of ACC deaminase. The bacterial culture was collected to determine the ACC deaminase activity by measuring the production of α-KB (the cleavage product of ACC) according Penrose and Glick (2003). Total protein concentrations were determined using a Bradford protein assay kit (Tiangen, Beijing, China). The ACC deaminase activity was expressed as the amount of α-KB produced per milligram of total protein per hour. The bacterial strain displaying the highest ACC deaminase activity was named JW-CZ2 and subjected to further investigations.

### Identification of JW-CZ2 by 16S Sequencing

Genomic DNA of JW-CZ2 was extracted using a Genomic DNA Extraction Kit (Sangon Biotech, Shanghai, China) following the manufacturer’s protocol. The universal forward primer (5′-CAGAGTTTGATCCTGGCT-3′) and reverse primer (5′-AGGAGGTGATCCAGCCGCA-3′) were used to amplify the 16S rDNA gene. PCR cycling was performed under the following conditions: 95°C for 5 min, 35 cycles of 94°C for 30 s, 60°C for 30 s, and 72°C for 90 s, with the final extension at 72°C for 8 min. The PCR product was sequenced by the Shanghai Sangon Biotech Co. Ltd. (Shanghai, China), and the data were deposited in the GenBank with the access number KP903465.

The 16S sequence was blasted against the GenBank database using the NCBI-BLASTn tool for species identification. Next, a phylogenic tree was constructed using the neighbor-joining method using MEGA 11 software with 1,000 bootstraps.

### Determination of Growth-Promoting Effects of JW-CZ2 on Tea Plants

*C. sinensis* seeds were obtained from a farm located in Henan Province, China. The seeds were surface-sterilized in 70% ethanol for 5 min, washed three times using sterile water, immersed in 0.5% NaClO solution for 10 min, and further washed five times in sterile water. One seed was sown in each plot (6 cm diameter, 12 cm height) filled with 500 g of acid-washed and autoclaved vermiculite (granule diameter 0.1–0.5 mm). The seeds were germinated, and then the seedlings were cultured in a plant growth chamber at 25 ± 2°C and 60–80% relative humidity. The photoperiod was 14 h:10 h with the light intensity of 4,000 lx. During the culture period, the seedlings were irrigated with sterile distilled water appropriately. After cultivation for 90 days, seedlings of similar shoot height and stem diameter were selected for inoculation of JW-CZ2.

JW-CZ2 strain was cultured in Luria–Bertani (LB) medium at 30°C and 200 rpm for 48 h. After centrifugation at 6,757 rpm for 10 min at 4°C, the cell pellets were washed and re-suspended in sterile saline with a bacterial density of approximately 3–5 × 10^8^ CFU/ml. For each seedling, 5 ml of bacterial suspension was inoculated using the root-drenching method (Ge et al., 2004), and the control seedlings received an equal volume of sterile saline. The treatment and the control group were repeated eight times independently. Afterward, plants were cultured in the growth chamber under the same condition for 180 days. Finally, seedlings were collected carefully from the pots and washed with tap water to remove soil particles. The shoot length and stem diameter were measured. After drying at 60°C until constant dry weight, the dry weights were determined.

### Effect of JW-CZ2 on Growth of Plant Pathogens

Six plant pathogens were selected for the tests, including *Fusarium oxysporum* f. sp. *cubense* (stored in Nanjing Agricultural University, China), *Diaporthe biconispora*, *Rhizoctonia solani*, *Fusarium oxysporum*, *Phomopsis macrospore*, and *Sphaeropsis sapinea* (stored in the Laboratory of Forest Pathology, Nanjing Forestry University, China). The growth-inhibition effects of JW-CZ2 on these pathogens were determined using the plate-confrontation method (Xie and Wu, 2021). First, a 5 mm hole was punched on the center of potato dextrose agar (PDA) agar plates using a sterile perforator. Second, the pathogens were inoculated in these holes, and JW-CZ2 was inoculated as streaks at 3 cm from the center of the plate. After being cultured at 28°C until the pathogens covered the whole dish in the control group, the width of inhibition zone in the experimental groups was measured using a vernier caliper. The width of inhibition zone is defined as the distance between the edge of the pathogen colony and the edge of the JW-CZ2 colony (mm). Each treatment was repeated four times.

### Effects of Inducing Factors on 1-Aminocyclopropane-1-Carboxylic Acid Deaminase Activity in JW-CZ2

To test the effects of single inducing factors on ACC deaminase activity, JW-CZ2 was cultured in 250 ml flasks containing 50 ml media. (1) JW-CZ2 was inoculated into DF salt minimal medium containing different ACC concentrations (0, 0.3, 3, 30, 300, and 3,000 μM) and then cultured at 28°C for 24 h; (2) JW-CZ2 was inoculated into DF salt minimal medium containing 3 mM ACC and then cultured at 20°C, 25°C, 28°C, 30°C, and 35°C for 24 h; (3) JW-CZ2 was inoculated into DF salt minimal medium containing 3 mM ACC at pH 6.5, 7.0, 7.5, 8.0, and 8.5 and then cultured at 28°C for 24 h; (4) JW-CZ2 was inoculated into DF salt minimal medium containing 3 mM ACC and then cultured at 28°C for 4, 8, 12, 16, 20, 24, 28, 36, and 40 h. At each time point or in each treatment, an aliquot was sampled from each flask to determine the ACC activity as described above. All experiments were performed in triplicates.

### Whole Genome Sequencing of JW-CZ2 and Genome Annotation

To further explore the mechanisms underlying the tea plant growth-promoting effects of JW-CZ2, the whole genome of JW-CZ2 was sequenced. Briefly, the genomic DNA of JW-CZ2 was isolated using the Wizard genomic DNA purification kit (Promega, Madison, WI). DNA quality and concentration were evaluated using the NanoDrop (Thermo Fisher Scientific, Wilmington, DE) and Qubit assay (Thermo Fisher Scientific, Wilmington, DE). The sequencing library was prepared using the SMRTbell Express Template Prep Kit 2.0 and then sequenced on a PacBio RS II sequencer following the manufacturer’s protocol (Pacific Biosciences, Menlo Park, CA, United States). Two single-molecule real-time (SMRT) cells were performed with a 120-min movie time per SMRT cell (Eid et al., 2009; Faino et al., 2015). SMRT Analysis portal version 2.1 was used for read filtering and adapter trimming with the default parameters, and 400 Mb of postfiltered data (approximately 80 × coverage) with an average read length of 7 kb were used for genome assembly. The genome was then *de novo* assembled using the Hierarchical Genome Assembly Process V3 (HGAP) incorporated in the SMRT Analysis package version 2.1 (Chin et al., 2013).

The gene structure of JW-CZ2 genome was predicted using the Glimmer Version 3.02 software, and the rRNA sequences were obtained using RNAmmer Version 1.2. To predict the biological functions, all genes were annotated by blasting against Non-Redundant (NR), Swiss-Prot, COG (clusters of orthologous groups of proteins), and KEGG (Kyoto Encyclopedia of Genes and Genomes) databases using the RAST annotation system (Tatusov et al., 2000; Moriya et al., 2007; Aziz et al., 2008; Overbeek et al., 2014; Brettin et al., 2015) with the-value cutoff of 1E–5.

### Determination of Phosphate Solubilization Ability of JW-CZ2

JW-CZ2 was inoculated in National Botanical Research Institute’s Phosphate (NBRIP) medium containing 0.5% Ca_3_(PO_4_)_2_, FePO_4_, or Mg_3_(PO_4_)_2_ as sole phosphate source. After incubation at 28°C and 180 rpm for 72 h, the culture liquid was centrifuged at 10,000 rpm at 4°C for 10 min, and the concentrations of soluble phosphate in the supernatant were determined using the molybdenum blue method following Watanabe and Olsen (1965).

### Determination of Indole Acetic Acid Production in JW-CZ2

JW-CZ2 was inoculated in King’s broth with or without 100 mg/L tryptophan and then cultured at 28°C and 200 rpm for 3 days. Each assay included 50 ml of culture media and was repeated three times. Next, the OD600 values of culture media were determined to evaluate cell densities. After centrifugation at 11,261 rpm for 10 min, 50 μl of supernatant was used to colorimetrically determine the production of IAA using the Salkowsky’s reagent (2% of 0.5 M FeCl_3_ in 35% HClO_4_ solution) (Benizri et al., 1998; Rahman et al., 2010). The data were normalized by setting OD600 as 1.

### Determination of Siderophore Production in JW-CZ2

To determine the production of siderophore, 1 μl of bacterial culture grown overnight in King’s broth was spotted on Chrome Azurol S agar plates (Ames-Gottfred et al., 1989). After incubation for 48 h at 28°C, the appearance of plates was observed by eye. The formation of an orange halo around bacterial colonies is a sign of siderophore production (Ali et al., 2014).

### Data Analyses

All data were analyzed using the SPSS 19.0 software for Windows (IBM Corporation, United States). Results are expressed as mean values ± standard deviation (SD). Effects of JW-CZ2 on the growth parameters of tea plants were compared between treatment and control using the Student’s *t*-test. The statistical significance between treatments and control in other experiments was assessed by one-way analysis of variance and Turkey’s test. *p* < 0.05 was considered statistically significant.

## Results and Discussion

### Isolation and Identification of Bacteria With 1-Aminocyclopropane-1-Carboxylic Acid Deaminase Activity

**FIGURE 1:**
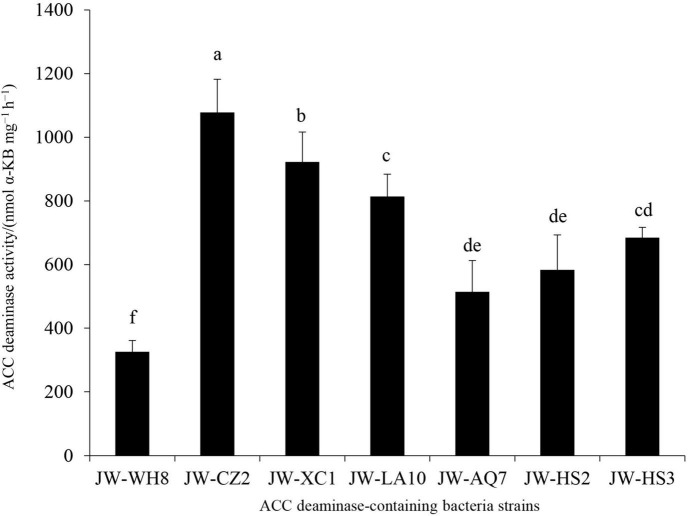
ACC deaminase activities of seven isolates from rhizosphere soil of *C. sinensis*. Data represent mean and SD (*n* = 3). Different letters indicate statistically significant differences (*p* < 0.05).

Since ACC deaminase was first isolated from *Pseudomonas* sp. strain ACP in 1978 (Honma and Shimomura, 1978), the production of ACC deaminase became a key trait for screening PGPR that facilitate plant growth under normal and/or adverse environmental conditions (Glick, 1995; Chandra et al., 2018; Gupta and Pandey, 2019). In this study, from the rhizosphere soils of tea plants, seven bacterial strains displayed significant ACC deaminase activity, which were named JW-WH8, JW-CZ2, JW-XC1, JW-LA10, JW-AQ7, JW-HS2, and JW-HS3. Among them, JW-CZ2 revealed the highest ACC deaminase activity (1,077.94 nmol α-KB mg^–1^ h^–1^), followed by JW-XC1 (922.65 nmol α-KB mg^–1^ h^–1^) and JW-AQ10 (813.70 nmol α-KB mg^–1^ h^–1^) (Figure 1). The strain JW-CZ2 was isolated from Qingyang County, Chizhou City, Anhui Province, China. The higher the ACC deaminase activity displays, the more valuable the strain is. Thus, we chose JW-CZ2 for further investigations.

**FIGURE 2:**
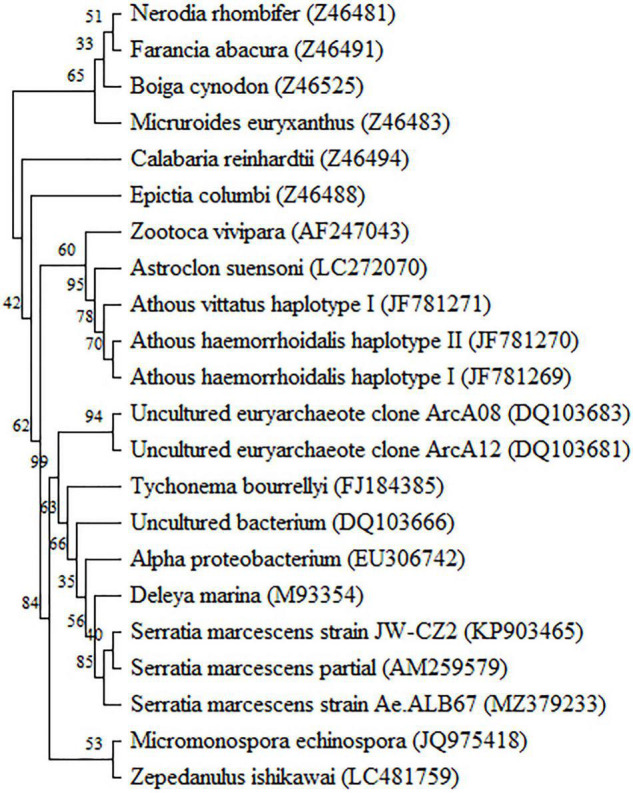
Phylogenetic tree based on the 16S rRNA sequences of J W-CZ2 and other bacterial strains. The GenBank accession numbers for nucleotide sequence data are shown at the beginning of each species.

Blasting against the GenBank database showed that the 1,503 bp partial 16S rDNA gene of JW-CZ2 was highly consistent to that of *Serratia marcescens* (KY379045) with an identity percentage of 99.93%. The phylogenetic tree also revealed that JW-CZ2 was clustered with two other *S. marcescens* strains (Figure 2). Thus, these results indicated that JW-CZ2 was a *S. marcescens* strain.

### Growth-Promoting Effects of *Serratia marcescens* JW-CZ2 on Tea Plants

In the present study, JW-CZ2 displayed a high ACC deaminase activity. Inoculation with JW-CZ2 for 180 days significantly increased shoot height, stem diameter, and dry weight of tea plants, with increasing rates of 53.44, 11.37, and 29.34%, respectively, in comparison to the control, indicating significant growth-promoting effects of *S. marcescens* JW-CZ2 on tea plants (Table 1). Similarly, Dhar Purkayastha et al. (2018) isolated *S. marcescens* strain ETR17 from the tea gardens in West Bengal and Assam, which also displayed promotive effects on tea plants. Forty-five days after inoculation, shoot length and root length increased approximately 20 and 32% compared to the control, respectively, which were lower than those of JW-CZ2 in the present study. Moreover, Chakraborty et al. (2013) reported the promotive effects of *S. marcescens* on tea plant growth with an increasing rate of approximately 50% for seedling height. Although other strains of *S. marcescens* have been reported, JW-CZ2 is a native strain to the Yangtze River region and might show better adaptability to the local environment. Taking these results together, *S. marcescens* is a plant growth-promoting bacterium (PGPB) to tea plants and displayed applicable values for tea plantation.

**TABLE 1:** Effects of inoculation with *S. marcescens* JW-CZ2 on growth parameters of tea seedlings.

Indices	Control	JW-CZ2	Rate of increase (%)
Shoot height (cm)	10.39 ± 0.38^a^	16.15 ± 0.43^b^	53.44
Stem diameter (mm)	2.11 ± 0.114^a^	2.35 ± 0.12^a^	11.37
Biomass (g)	8.52 ± 0.27^a^	11.02 ± 0.27^b^	29.34

*Different letters in the same rows indicate significant differences (p < 0.05).*

### Effects of Culture Conditions on 1-Aminocyclopropane-1-Carboxylic Acid Deaminase Activity in JW-CZ2

**FIGURE 3:**
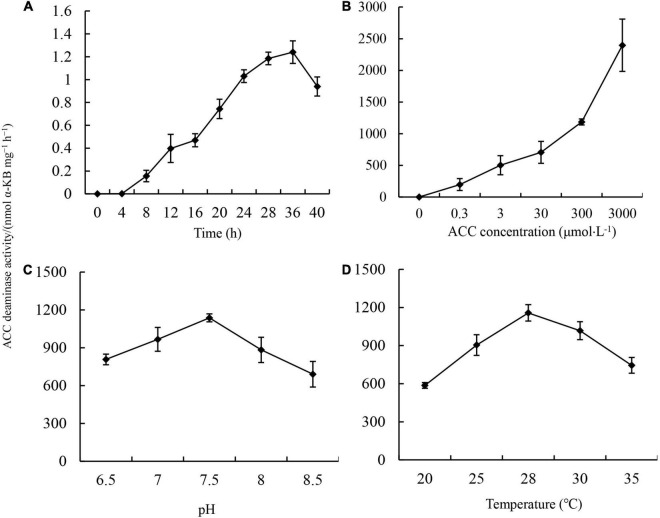
Effects of inducing time **(A)**, ACC concentration **(B)**, pH **(C)**, and inducing temperature **(D)** on ACC deaminase activity in *S. marcescens* JW-CZ2. Data indicate mean and standard deviation (*n* = 3). Different letters indicate significant differences (*p* < 0.05).

The major mechanism underlying the promotion of PGPR with ACC deaminase activity on plant growth is the repression of ethylene production in plants (Penrose and Glick, 2003). ACC deaminase is an inducible enzyme. To further explore the applicable potential of JW-CZ2 as a biofertilizer, the effects of inducing time, ACC concentration, pH, and temperature on ACC deaminase activity were investigated (Figure 3).

In the absence of ACC, ACC deaminase activity was almost not detected in the JW-CZ2. However, after addition of 3 mM ACC, ACC deaminase activity increased from 4 to 36 h and then started to decrease at 40 h. The highest activity was 1,240.38 nmol α-KB mg^–1^ h^–1^, which was observed at 36 h (Figure 3A). With increasing ACC concentrations from 0.3 to 3,000 μM in culture media, ACC activity increased greatly and significantly, suggesting that JW-CZ2 could adapt to various concentrations of ACC (Figure 3B). JW-CZ2 showed significant ACC deaminase activity from pH 6.5 to 8.5 and with the highest ACC activity at pH 7.5 (Figure 3C). From 20 to 35°C, JW-CZ2 functioned well to produce ACC deaminase, and the maximum activity was observed at 28°C (Figure 3D). Similarly, *in vitro* expressed ACC deaminase from *Enterobacter cloacae* and *Enterobacter cancerogenus* also revealed activity at pH ranging from 5.5 to 8.5 and temperature ranging from 4 to 50°C, with the highest ACC activity at pH 7.5 and at 28°C (Jha et al., 2012). The bacterial strain XG32 revealed the highest ACC activity at 25°C, pH from 7.0 to 8.0, and inducing time of 24 h (Shen et al., 2008). Overall, these data indicated that JW-CZ2 could grow well and produce ACC deaminase activity at conditions with a broad range of ACC concentration, pH, and temperature, which increased the applicable potential of JW-CZ2 in fields as a biofertilizer.

### Genome Sequencing of *Serratia marcescens* JW-CZ2

The genome of JW-CZ2 consisted of 4,925,622 bp with Guanine-Cytosine (GC) content of 59.82%. The DNA coding sequences were 4,268,136 bp in total, which contained approximately 4,670 coding sequences (CDS), including 4,547 proteins, 96 tRNA genes, and 22 rRNA genes. Among the CDS, 4,197 (89.83%) were successfully annotated to at least one database, while 475 (10.17%) were unknown (Table 2). On the other hand, Khan et al. (2017) reported that the genome of *S. marcescens* RSC-14 was 5.12 Mbp in length and included 4,593 CDS, 22 rRNA, and 88 tRNA genes. Obviously, the strain JW-CZ2 identified in the present study was different from RSC-14.

**TABLE 2:** Statistics of *S. marcescens* JW-CZ2 genome sequencing.

Features	Value
Genome size (bp)	4,925,622
Contig numbers	1
DNA coding (bp)	4,268,136
G + C%	59.82
DNA G + C (bp)	2,946,449
CDS	4,670
Proteins	4,547
tRNA number	96
rRNA number	22
ncRNA number	271

**FIGURE 4:**
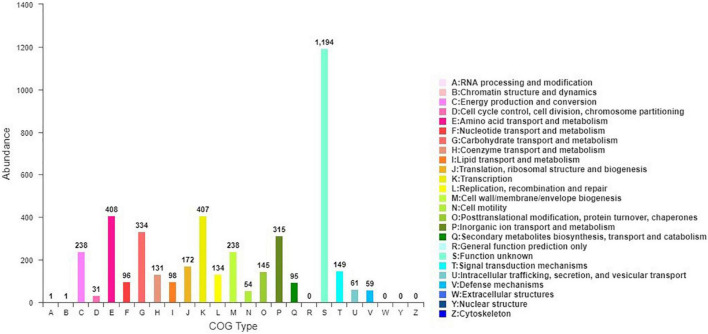
Clustering of orthologous genes (COG) of predicted genes in JW-CZ2 genome.

Blasting against the COG database revealed that 4,274 of the 4,670 predicted CDS (91.52%) could match to at least one COG category. Among them, the top four were metabolism of amino acid transport and metabolism (9.54%), transcription (9.52%), carbohydrate transport and metabolism (7.81%), and inorganic ion transport and metabolism (7.37%). In addition, 27.94% of the predicted CDS were still functionally unknown (Figure 4).

**FIGURE 5:**
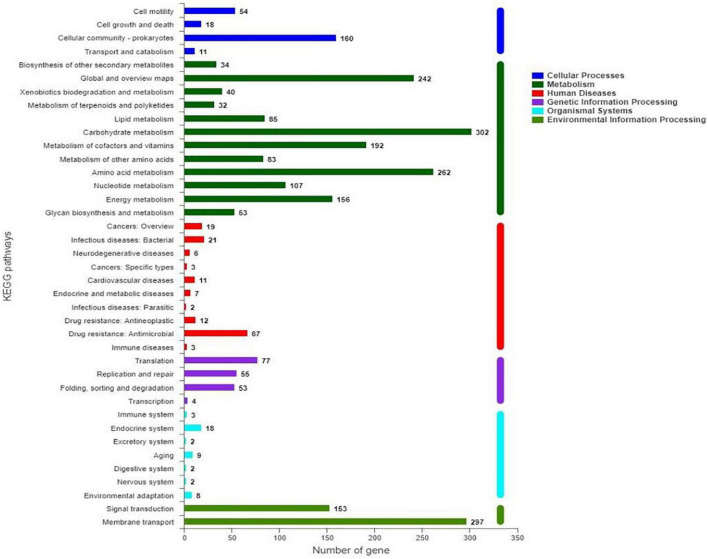
Classification of KEGG pathways of predicted genes in *S. marcescens* JW-CZ2 genome.

Characterization of KEGG pathways showed that 2,937 genes (62.89% of all CDS) were involved in at least one pathway (Figure 5). Among them, the top four pathways were carbohydrate metabolism, membrane transport, amino acid metabolism, and global and overview maps, in total representing 23.62% of the predicted CDS. In addition, metabolism of cofactors and vitamins, energy metabolism, and signal transduction included 4.11, 3.34, and 3.28% of CDS, respectively.

### Genes Related to Plant Growth-Promoting Properties

The protein sequence of ACC deaminase (GenBank access number: WP_195315725.1) was used to blast against the genome of JW-CZ2, and the most similar sequence was D-cysteine desulfhydrase (SC_2041, Table 3). Todorovic and Glick (2008) reported that ACC deaminase and D-cysteine desulfhydrase could interconvert after directed mutagenesis. Thus, we speculated that D-cysteine desulfhydrase in JW-CZ2 might function as an ACC deaminase.

**TABLE 3:** Major genes related to plant growth-promoting effects of JW-CZ2.

Annotation entry	Gene name	Gene description

ACC deaminase activity		
SC_2041	*dcyD*	D-Cysteine desulfhydrase

Phosphate solubilization		
SC_2575	*pqqB*	Pyrroloquinoline quinone biosynthesis protein B
SC_2576	*pqqC*	Pyrroloquinoline quinone biosynthesis protein C
SC_2577	*pqqD*	Coenzyme PQQ synthesis protein D
SC_2578	*pqqE*	Pyrroloquinoline quinone biosynthesis protein PqqE
SC_0129	*phoU*	Phosphate transport system regulator PhoU
SC_0130, 1348	*pstB*	Phosphate ABC transporter ATP-binding protein
SC_0131	*pstA*	Phosphate transporter permease subunit PtsA
SC_1347	*pstA*	Phosphate ABC transporter, permease protein PstA
SC_0132	*pstC*	Phosphate transporter permease subunit PstC
SC_1346	*pstC*	Phosphate ABC transporter permease
SC_0133, 3993	*pstS*	Phosphate ABC transporter substrate-binding protein
SC_0540	*Edd*	Phosphogluconate dehydratase
SC_3713	*Pgl*	6-Phosphogluconolactonase

**IAA biosynthesis**		

SC_1476	*ipdC*	Indolepyruvate decarboxylase
SC_4042	–	Indolepyruvate decarboxylase

**Siderophore production**		

SC_3905	*hemH*	Ferrochelatase
SC_0490	*Bfr*	Bacterioferritin

As reported, genes in relation to phosphate solubilization and IAA production were observed in the genome of *S. marcescens* RSC-14, which were believed to be major reasons for the plant growth-promoting function of RSC-14 (Khan et al., 2017). In the genome of JW-CZ2, similar results were found. Gluconic acid is an important molecule contributing to dissolving inorganic phosphate (Bagchi et al., 1997; Holguin and Glick, 2001). Gluconic acid synthesis requires pyrroloquinoline quinone (PQQ) as cofactor, which is produced by PQQ biosynthesis proteins (Vessey, 2003). In the present study, four genes encoding PQQ biosynthesis proteins B, C, D, and E (*pqqB*, *pqqC*, *pqqD*, and *pqqE*) were found in the genome of JW-CZ2 (Table 3). In addition, phosphate transporters are specific proteins responsible for inorganic phosphate uptake (Hoffer et al., 2001). In the present study, six genes encoding four phosphate transporters (*pstB*, *pstA*, *pstC*, and *pstS*) were observed in the JW-CZ2 genome (Table 3), suggesting an enhancive functional basis of phosphate uptake in JW-CZ2. These results predicted that JW-CZ2 might display a strong capacity of phosphate solubilization and transportation, which might be reasons to explain its plant growth-promoting function. In addition, two genes encoding indolepyruvate decarboxylase (*ipdC*) were observed in the genome of JW-CZ2 (Table 3), which are key genes for IAA biosynthesis, converting indole-3-pyruvate to indole-3-ethanol (Brandl and Lindow, 1996). These results suggested that JW-CZ2 might be able to synthesize IAA to stimulate plant growth.

Another *S. marcescens* strain was reported to produce siderophore, which is a typical plant growth-promoting trait (Baruah and Handique, 2014). Ferrochelatase (*hemH*) is an enzyme to sequestrate iron (Cornelis et al., 2011), and bacterioferritin (*bfr*) incorporates iron to form siderophore (Expert et al., 2008). In the present study, both *hemH* and *bfr* were observed in the JW-CZ2 genome, suggesting its potential to produce siderophore. In comparison, genes associated with siderophore was not reported in the genome of *S. marcescens* RSC-14 (Overbeek et al., 2014).

### Validation of Mechanisms Underlying the Promotion of *Serratia marcescens* JW-CZ2 on Tea Plant Growth

Based on the genome sequencing, we speculated that the capacity of phosphate solubilization and production of IAA and siderophore might be potential mechanisms underlying the promotion of *S. marcescens* JW-CZ2 on tea plant growth. In the present study, after addition of Ca_3_(PO_4_)_2_, FePO_4_, and Mg_3_(PO_4_)_2_ in the culture media for 72 h, 806.06 ± 24.15, 42.89 ± 3.27, and 48.84 ± 2.96 mg/L soluble phosphate was detected in the culture media (Table 4), demonstrating the phosphate solubilization capacity of JW-CZ2, which was consistent with the prediction from genome sequencing of both *S. marcescens* JW-CZ2 in the present study and *S. marcescens* RSC-14 in Khan et al. (2017) and experimental evidence for *S. marcescens* PH1 and PH2 in Mohamed et al. (2018). In comparison, the phosphate solubilization capacity of JW-CZ2 was higher than that of *Bacillus* sp. TRSB16, which could solubilize 239 mg/L phosphate in the presence of Ca_3_(PO_4_)_2_ (Banerjee, 2010), but lower than that of *S. marcescens* GPS-5 mutants [higher than 1,500 mg/L phosphate in the presence of Ca_3_(PO_4_)_2_; Tripura et al., 2007]. P is a limiting element for plants. The phosphate solubilization capacity of *S. marcescens* JW-CZ2 could convert insoluble inorganic P to soluble status, which should increase P availability and promote plant growth.

**TABLE 4:** Phosphate solubilization by the JW-CZ2 strain.

Ca_3_(PO_4_)_2_	FePO_4_	Mg_3_(PO_4_)_2_
806.06 ± 24.15	42.89 ± 3.27	48.84 ± 2.96

*Data indicate soluble phosphate concentration in media (mg/L).*

**FIGURE 6:**
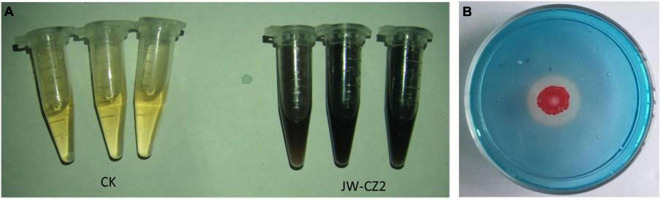
IAA **(A)** and siderophore production **(B)** by JW-CZ2.

After culture in the presence of tryptophan for 3 days, 7.27 ± 0.24 μg/ml IAA was detected in the culture media. In addition, on the Chrome Azurol S agar plate, JW-CZ2 formed an orange halo around the colonies, indicating the production of siderophore (Figure 6). These results were consistent with the genome sequencing of JW-CZ2. Partially similarly, IAA production was observed in *S. marcescens* AEP5, AEN38, and AEP66 strains, while siderophore was detected in *S. marcescens* AEP85 strain (Aktan and Soylu, 2020). IAA is a phytohormone and promotes plant growth at certain concentrations. Bacterial siderophore is an important mechanism for iron uptake in plants (Yehuda et al., 1996), which could convert biologically unavailable Fe element to available form adsorbed by plants (Katiyar and Goel, 2004). Thus, production of IAA and siderophore might also contribute to the tea plant-promoting effects of *S. marcescens* JW-CZ2.

### Inhibitory Effects of *Serratia marcescens* JW-CZ2 on Plant Pathogens

**FIGURE 7:**
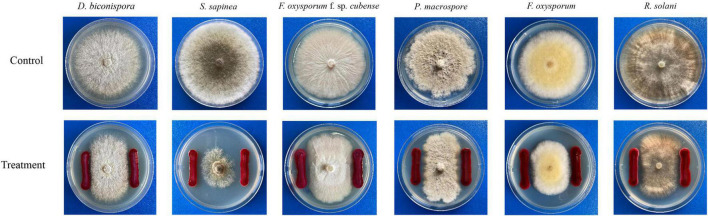
The results of confrontation culture between *S. marcescens* JW-CZ and six different plant pathogens.

As shown in Table 5 and Figure 7, JW-CZ2 showed significant inhibitory effects on all the six tested plant pathogens, and the inhibitory effect was the most significant on *P. macrospore* and *S. sapinea*, with width of inhibition zones of 3.22 ± 0.81 and 3.17 ± 0.98 mm, respectively. *P. macrospore* is an important pathogenic fungus and displayed significant inhibition to tea plant growth (Ponmurugan and Baby, 2007). *S. sapinea* causes tip blight and death in *Pinus radiata* (Corrêa et al., 2012). These results indicated that inhibition by JW-CZ2 of plant pathogenic fungi might be a potential reason to explain its plant growth-promoting effects.

**TABLE 5:** The inhibitory effect of *S. marcescens* strain JW-CZ2 on six different plant pathogens.

Pathogens	Inhibition zone width (mm)
*D. biconispora*	2.51 ± 0.87
*S. sapinea*	3.17 ± 0.98
*F. oxysporum* f. sp. *cubense*	2.97 ± 0.86
*P. macrospore*	3.22 ± 0.81
*F. oxysporum*	1.73 ± 0.65
*R. solani*	2.08 ± 0.71

## Conclusion

From rhizosphere soils of tea plants, we identified *S. marcescens* strain JW-CZ2 which displayed high ACC deaminase activities at various ACC concentrations, pH, and temperature. Application of this strain significantly promoted the growth of tea seedlings. Genome sequencing and experimental evidences indicated that JW-CZ2 could produce IAA and siderophore, enhance the solubilization of inorganic phosphate, and inhibit the growth of pathogenic fungi, which all contributed to its promotive functions of plant growth. Overall, *S. marcescens* JW-CZ2 is a promising tea plant growth-promoting bacterium.

## Data Availability Statement

The datasets presented in this study can be found in online repositories. The names of the repository/repositories and accession number(s) can be found in the article/Supplementary Material.

## Author Contributions

HL and J-HR designed the experiments. G-HC, J-JS, and SC collected the samples. HL and YF performed the genomic analysis and analyzed the data. HL drafted the manuscript. All authors revised the manuscript.

## Conflict of Interest

The authors declare that the research was conducted in the absence of any commercial or financial relationships that could be construed as a potential conflict of interest.

## Publisher’s Note

All claims expressed in this article are solely those of the authors and do not necessarily represent those of their affiliated organizations, or those of the publisher, the editors and the reviewers. Any product that may be evaluated in this article, or claim that may be made by its manufacturer, is not guaranteed or endorsed by the publisher.
