# Supplementary material for: Isolation, Characterization, and Tea Growth-Promoting Analysis of JW-CZ2, a Bacterium With 1-Aminocyclopropane-1-Carboxylic Acid Deaminase Activity Isolated From the Rhizosphere Soils of Tea Plants
Source: Front Microbiol. 2022 Feb 28;13:792876. doi: 10.3389/fmicb.2022.792876 (PMC8918981; doi:10.3389/fmicb.2022.792876)
Supplement: Supplementary file 1 [file Table_1.DOCX]

**Table S1 The soil sample of rhizosphere of *Camellia sinensis* in Anhui tea region**

| **Number** | **Sampling points** | **tea region** | **Coordinates** |
| --- | --- | --- | --- |
| 1 | Yuexi County, Anqing City | Tea-area of Ta-pieh Mountains | N30°56′24.28′′ E116°20′8.34′′ |
| 2 | Qingyang County, Chizhou City | Tea-area of Ta-pieh Mountains | N30°31′49.49′′E117°45′55.01′′ |
| 3 | Tunxi District, Huangshan City, | Tea-area of Mount Huangshan | N29°42′47.42″ E118°15′36.49″ |
| 4 | Shucheng County, Lu'an City | Tea-area of Mount Huangshan | N31°26′52.33″ E116°52′10.19′′ |
| 5 | Jixi County, Xuancheng City | Tea-area of Chiang-nan Hilly Region | N30°04′11.89′′E118°32′34.17′′ |
| 6 | Xuanzhou District, Xuancheng City | Tea-area of Chiang-nan Hilly Region | N30°58′49.01″ E118°41′41.13″ |
| 7 | Wuwei County, Wuhu City | Tea-area of Yangtze-Huaihe | N31°20′28.59′′ E117°55′5.29′′ |
